# Poly (ADP-Ribose) Polymerase-1 (PARP1) Deficiency and Pharmacological Inhibition by Pirenzepine Protects From Cisplatin-Induced Ototoxicity Without Affecting Antitumor Efficacy

**DOI:** 10.3389/fncel.2019.00406

**Published:** 2019-09-10

**Authors:** Anke Tropitzsch, Marcus Müller, François Paquet-Durand, Frank Mayer, Hans-Georg Kopp, Andre Schrattenholz, Andrea Müller, Hubert Löwenheim

**Affiliations:** ^1^Department of Otorhinolaryngology, Head and Neck Surgery, Tübingen Hearing Research Center, University of Tübingen Medical Center, Tübingen, Germany; ^2^Cell Death Mechanisms Lab, Institute for Ophthalmic Research, University of Tübingen, Tübingen, Germany; ^3^Department of Oncology, Hematology, Immunology, Rheumatology and Pulmology, University of Tübingen Medical Center, Tübingen, Germany; ^4^ProteoSys AG, Mainz, Germany

**Keywords:** cisplatin, ototoxicity, otoprotection, organ culture, PARP, hair cell loss, inner ear, PARP-inhibitor

## Abstract

Cisplatin remains an indispensable drug for the systemic treatment of many solid tumors. However, a major dose-limiting side-effect is ototoxicity. In some scenarios, such as treatment of germ cell tumors or adjuvant therapy of non-small cell lung cancer, cisplatin cannot be replaced without undue loss of efficacy. Inhibition of polyadenosine diphosphate-ribose polymerase-1 (PARP1), is presently being evaluated as a novel anti-neoplastic principle. Of note, cisplatin-induced PARP1 activation has been related to inner ear cell death. Thus, PARP1 inhibition may exert a protective effect on the inner ear without compromising the antitumor activity of cisplatin. Here, we evaluated PARP1 deficiency and PARP1 pharmacological inhibition as a means to protect the auditory hair cells from cisplatin-mediated ototoxicity. We demonstrate that cisplatin-induced loss of sensory hair cells in the organ of Corti is attenuated in PARP1-deficient cochleae. The PARP inhibitor pirenzepine and its metabolite LS-75 mimicked the protective effect observed in PARP1-deficient cochleae. Moreover, the cytotoxic potential of cisplatin was unchanged by PARP inhibition in two different cancer cell lines. Taken together, the results from our study suggest that the negative side-effects of cisplatin anti-cancer treatment could be alleviated by a PARP inhibition adjunctive therapy.

## Introduction

Cisplatin [cis-diammin dichloroplatinum (II)] belongs to the essential cytotoxic drugs for the treatment of solid tumors. Applications include a wide range of cancers in children and adults. The introduction of cisplatin has increased the cure rate of metastatic germ cell tumors in an unprecedented way from approximately 20% in the era before cisplatin to 80–90% with cisplatin-based combination chemotherapy. Nephrotoxicity (renal dysfunction) and bone marrow toxicity (anemia, neutropenia, and thrombocytopenia), together with ototoxicity, are among the major dose-limiting side-effects of cisplatin (Ekborn et al., 2003). Ototoxic symptoms persisted in 20% of adult testicular cancer patients (median age of 31 years) but may reach prevalence over 50% in patients receiving cumulative doses of cisplatin above 400 mg/m^2^ (Bokemeyer et al., 1998). About 62% of children and adolescents treated with platinum-based chemotherapy acquired bilateral hearing loss in the conventional audiometric frequency range. This number increased to 81% for a decrease in evoked distortion product otoacoustic emissions (DPOAE) amplitudes and dynamic ranges. When extended high frequency (EHF) range audiometry was applied, functional deficits increased further to 94% (Knight et al., 2007). These data from sensitive audiological tests suggest that cisplatin-induced ototoxicity is more frequent than measured by conventional audiometry (Knight et al., 2007).

Cisplatin ototoxicity is mediated by reactive oxygen species, including superoxide anions (Dehne et al., 2001; Rybak et al., 2007), and transcription factors such as NF-κB have been suggested as further cell death mediators (Rybak et al., 2007). However, the damage caused by cisplatin to cells cannot solely be explained through DNA interaction (Sherman et al., 1985) but also by the cell’s ability to detect and respond to DNA damage (Kerr et al., 1994).

One important DNA repair mechanism is activation of poly (ADP-ribose) polymerase-1 (PARP1). Even 50 years after Paul Mandel first described a nuclear enzymatic activity that synthesizes an adenine-containing RNA-like polymer (Chambon et al., 1963), PARP1 and its activation mechanism in the ear have not been thoroughly investigated. PARP1 detects and marks damaged DNA sequences and acts as scaffolding protein to orchestrate other factors in the DNA repair pathway. Similar as many other DNA repair mechanisms, its action can have two opposing effects (Rouleau et al., 2010): (1) Sufficient repair of the damage can allow for survival of the cell, while (2) in cases of more severe damage, the failed efforts to repair the damage can result in fatal ATP depletion and cell death. The poly-ADP ribosylation, which is primarily catalyzed through PARP1, is a post-translational modification of nuclear proteins where NAD^+^ is used as a substrate (Burkle, 2005). Strong up-regulation of PARP1 can be observed in the early stages of cell death (Yu et al., 2002). This mechanism plays a crucial role in several pathophysiological processes and can be therapeutically exploited through the use of PARP1 inhibitors (Virag and Szabo, 2002), including in the inner ear, where the PARP1 inhibitor 3-aminobenzamide (3-AB) has been shown to be otoprotective (Tabuchi et al., 2001, 2003). In other sensory systems, such as the retina, PARP1 gene knock-out increases resistance to retinal degeneration (Sahaboglu et al., 2010), and PARP inhibitors, including the anti-cancer drug olaparib, improve retinal viability (Sahaboglu et al., 2016).

Recently, more evidence was presented that ototoxicity of cisplatin can be influenced through the regulation of PARP1 (Kim et al., 2014, 2016). It was reported that a decrease in SIRT1 activity and expression facilitated increased PARP1 activity and aggravated cisplatin-mediated ototoxicity. The expression level and activity of SIRT1 in turn was suppressed by the reduction of intracellular NAD^+^ levels. Hyperactivation of PARP1 led to depletion of NAD^+^ and ATP production, followed by cochlear cell death. Restoring cellular NAD^+^ levels by application of β-lapachone or dunnione as substrates for NAD(P)H dehydrogenase quinone-1 (NQO1) promoting conversion of NADH to NAD^+^ prevented the toxic effects of cisplatin. These results suggest that direct modulation of cellular NAD^+^ levels by pharmacological agents could be a promising therapeutic approach for protection from cisplatin-induced ototoxicity (Kim et al., 2014). Thus, PARP-1 inhibition may provide for otoprotection during cisplatin treatment. Here, we tested pirenzepine and several other, well-validated PARP inhibitors of various potencies. The prototypical PARP inhibitor 3-AB has an EC50 of 200 μM. The structure of 3-AB is similar to that of NAD^+^ so that it binds to PARP and prevents it from using up NAD^+^. Another PARP inhibitor is PJ34, a cell-permeable, water-soluble phenanthridinone-derivative that is a 10.000-fold more potent PARP inhibitor with an EC50 of 20 nM. DPQ is also a potent and selective inhibitor of PARP (EC50 = 40 nM). PARP inhibition is a general neuroprotective principle, likely attenuating the intrinsic, i.e., mitochondrial pathway of apoptosis, which can be induced by a variety of pathological or toxic conditions (Schrattenholz and Soskic, 2006). Pirenzepine (Gastrozepin) is an approved muscarinic M1 receptor selective antagonist (Del Tacca et al., 1989) that reduces gastric acid secretion and muscle spasms and is used in the treatment of peptic ulcers. It is also investigated for use in myopia (Mak et al., 2018). The PARP1 inhibitor pirenzepine would allow for a direct entry into clinical development and use after a “proof of concept” on the basis of drug repositioning, as pirenzepine is an active substance already on the market with a different indication area and mechanism of action. For further development an already identified metabolite (LS-75) with a highly specific effect on PARP1 is available. LS-75 is a metabolite of pirenzepine (Riss et al., 2009). *In vivo*, pirenzepine is metabolized to 5,11-dihydro-benzo[e]pyrido[3,2-b][1,4]diazepin-6-one, tagged LS-75 (Schrattenholz, 2006; Schrattenholz and Soskic, 2006).

Otoprotective drug screening for the prevention of cisplatin-induced ototoxicity unfortunately is impeded by the high mortality encountered in animal models due to the systemic toxicity of cisplatin that may precede ototoxic effects. This might be reflected in the many different protocols utilizing single (Schmitt and Rubel, 2013) or multiple (Roy et al., 2013) cisplatin applications (see also Tropitzsch et al., 2014 for review). Toward the development of an *in vivo* model, a quantitative assay employing the lateral line of zebrafish larvae was introduced to facilitate drug screening for ototoxic and otoprotective agents (Ton and Parng, 2005; Ou et al., 2007). The model provided evidence of dose-dependent cisplatin-induced hair cell loss. This limitation may be overcome by the development of validated *in vitro* models. Previous *in vitro* models to study cisplatin ototoxicity in the mammalian cochlea utilized tissue culture techniques involving isolated tissue fragments dissected from the postnatal inner ear organ (Liu et al., 1998; Zhang et al., 2003; Yarin et al., 2005; Previati et al., 2007).

In the present study, we utilized a rotating bioreactor culture system under simulated microgravity conditions (Hahn et al., 2008; Arnold et al., 2010; Tropitzsch et al., 2014). The system allows for maintaining the entire postnatal mouse inner ear organ in culture for up to seven days. In this model, controlled sensory cell lesions can be induced by ototoxic agents as toxicological models of hair cell degeneration and hair cell loss. We use this model to show that both genetic inactivation of PARP1 and pharmacological inhibition of PARP protects inner and outer hair cells from cisplatin induced damage.

## Materials and Methods

### Animals

PARP1-deficient mice (Wang et al., 1995) and wild-type 129SV mice used in this study were obtained from an in-house breeding colony maintained in a specified pathogen free (SPF) animal facility. Animal use for organ explants was approved by the Committee for Animal Experiments of the Regional Council (Regierungspräsidium) of Tübingen. PARP1 deficient mice were originally purchased from Jackson Laboratories (Parp1^TM 1*Zqw*^; Mouse Strain Datasheet – 002779) and bred with 129SV wildtype obtained from the Leibniz Institute on Aging – Fritz Lipmann Institute (FLI; Prof. ZQ Wang, Jena, Germany). Genotyping was performed on each animal by PCR (according to JAX Standard PCR protocol, 2003; primers CCA GCG CAG CTC AGA GAA GCC A for Wildtype; CAT GTT CGA TGG GAA AGT CCC) for common; AGG TGA GAT GAC AGG AGA TC for mutant).

### Cell Culture System Setup

Details of the methods are given in Hahn et al. (2008), Arnold et al. (2010), Tropitzsch et al. (2014). In short, explants of the inner ear organ of mice at postnatal day 7 were obtained. Animals had a bodyweight of 5–6.5 g. The explants were cultured in 55-ml HARV culture vessels mounted on a Rotary Cell Culture System (RCCS^TM^-4; Synthecon Inc., Houston, TX, United States). All dissections were performed in a laminar flow cell culture hood under sterile conditions. After decapitation, the complete inner ear bony labyrinth capsules were dissected from the skull base in ice-cold Hank’s balanced saline solution (HBSS) supplemented with 10 mM HEPES buffer (pH 7.3). The perilymphatic fluid spaces were opened via micro-dissection to provide access of the culture medium to the inner ear sensory epithelia. Immediately after completing the micro-dissection, inner ear organs were inoculated in a HARV vessel filled with pre-warmed culture medium, and rotation was started for 24 h at a rotation speed of 25–35 rounds per minute (RPM). The bioreactor was placed in a 37°C humidified 5% CO_2_/95% air incubator. The culture medium was Neurobasal^TM^ Medium (Invitrogen, Inc., Carlsbad, CA, United States) supplemented with 1 x B27 supplement (Invitrogen, Inc.), 5 mM glutamine, 10 mM HEPES (Invitrogen, Inc.), and 100 U penicillin (Sigma, St. Louis, MO, United States).

Cisplatin (MW: 300.05 g/mol, Sigma, St. Louis, MO, United States) was applied to the culture medium at concentrations ranging from 0.1 to 5 μg/ml for 24 h. After termination of the culture, inner ear explants were fixed with 4% paraformaldehyde, and the organ of Corti was micro-dissected as a whole mount preparation and divided into basal, middle, and apical segments. The surface morphology of the organ of Corti, in particular hair cell stereocilia bundles, were visualized by Phalloidin labeling of F-actin and immunohistochemical labeling for myosin VIIa. Whole mount specimens were washed with phosphate-buffered saline (PBS), permeabilized with 0.1% Triton X-100 in PBS for 5 min, and incubated with Phalloidin conjugated to the dye Alexa488 (Molecular Probes Inc., Eugene, OR, United States) for 20 min in the dark at room temperature. After washing in PBS, the whole mount specimens were mounted using Vectashield-DAPI (Vector Laboratories, Burlingame, CA, United States).

### Quantification of Sensory Hair Cell Maintenance and Statistical Analysis

Sensory hair cell maintenance and hair cell loss were quantified for organ preservation and toxicity assays (Tropitzsch et al., 2014). The effects of these experimental paradigms were quantified in whole mount preparations of the organ of Corti. These preparations consisted of three or four segments of the organ of Corti, designated basal, middle, and apical segments. The whole mount preparations were analyzed using a Zeiss Axioplan 2 epifluorescence microscope. Photomicrographs were taken and segments analyzed off-line. The length of each cochlea segment was determined at the midline between the inner and outer hair cell area along the longitudinal axis of the organ of Corti. A software module (Soft Imaging System, Stuttgart, Germany) allowing curved length measurements was used. Total cochlear length was determined by adding the length values of each segment. The inner hair cells and outer hair cells were counted using the ImageJ (Abramoff et al., 2004) plugin “Cellcounter,” which also allows to store the coordinates of the cells. These coordinates were used to determine the longitudinal position along the cochlear duct to allow for the construction of cochleograms (custom made program). Cisplatin induced hair cell loss showed a marked base to apex gradient with the base being more vulnerable. Hair cell preservation was quantified as the fraction of cochlear length with less than 10% hair cell loss, as seen in the cochleogram. All values of length measurements are presented as the mean fraction ± *SD* for the inner ear organ preservation assay. Differences between experimental groups were assessed using JMP (Version 14, SAS Institute). *t*-tests were performed to assess significance (^∗^*p* < 0.05, ^∗∗^*p* < 0.01 and ^∗∗∗^*p* < 0.001 values were considered significant), multiple testing was accounted for using a Dunnett’s test, comparing a number of treatments/conditions with a single control. SigmaPlot (Version 8, SPSS Inc.) was used to fit a typical dose-response curve with a variable slope parameter (four Parameter Logistic Equation) to obtain a median effective concentration (EC50):

(1) $$y=\min+\frac{\max-\min}{1+{\left(\frac{x}{E\,C\,50}\right)}^{H\,i\,l\,l\,s\,l\,o\,p\,e}}$$

where min is the bottom of the curve, max is the top of the curve, EC50 is the median effective concentration, and Hillslope characterizes the slope of the curve at its midpoint.

### Cell Culture Treatments and Analysis

A 3-(4,5-dimethylthiazol-2-yl)-2,5-diphenyltetrazoliumbromide (MTT) assay was performed as previously described (Sieuwerts et al., 1995). Two established metastatic germ cell tumors cell lines derived from human embryonal carcinomas NTera2 (NT2) and 2102 EP were used. NTera-2 cells (NT2/D1, ATCC CRL-1973 cells) were purchased from the American Type Tissue Culture (ATCC). 2102 EP cells were a kind gift of Dr. Looijenga, Erasmus Medical Center Rotterdam (Wang et al., 1981).

In brief, the cell lines NT2 and 2102 EP were rinsed with PBS (Biochrom), trypsinised and re-suspended in 1 ml of the appropriate culture medium to count the cells in a hemocytometer chamber. A total of 4 × 10^3^ cells/well were seeded in 96-well. Cells were allowed to adhere overnight. The cells were exposed to drugs (cisplatin or a test drug) for up to 24 h. The medium was then removed, and 0.2 ml MTT solution (final concentration: 0.5 mg/mL MTT; Sigma) was added. The plates were incubated for 2 h, the medium was removed, 0.1 ml DMSO was added, the plates were agitated for 15 min, and the optical density was read using a photometer (MRX Revelation, Dynex Technologies, VWR International, Bruchsal, Germany) at 570 nm. The optical density readout was averaged across wells, corrected for the water-value, and expressed as the percent survival relative to untreated cells. Error bars were calculated using error propagation for divisions (fractional uncertainties add in quadrature). The PARP inhibitors and their respective concentrations: Pirenzepine (10 μM), LS-75 (10 μM), 3-Aminobenzamide (3-AB, 100 μM), DPQ (1 μM), PJ34 (1 μM), and 0.1% DMSO (Pirenzepine and LS-75 were obtained from ProteoSys, 3-AB, PJ34, DPQ from Sigma). Pirenzepine (11-[2-(4-methyl-piperazin-1-yl)-acetyl]-5,11-dihydro-benzo[e]pyrido[3,2-b][1,4]diazepin-6-one) was originally developed as M1 selective muscarinergic antagonist. Pirenzepine is used as gastric acid secretion inhibitor worldwide and was re-introduced as potential neuroprotective compound via PARP1 modulation. *In vivo*, it is metabolized to 5,11-dihydro-benzo[e]pyrido[3,2-b][1,4]diazepin-6-one, also known as LS-75. This compound was previously found to be an inhibitor of PARP1 (Riss et al., 2009).

## Results

### Cisplatin Dose-Response Curve

**FIGURE 1:**
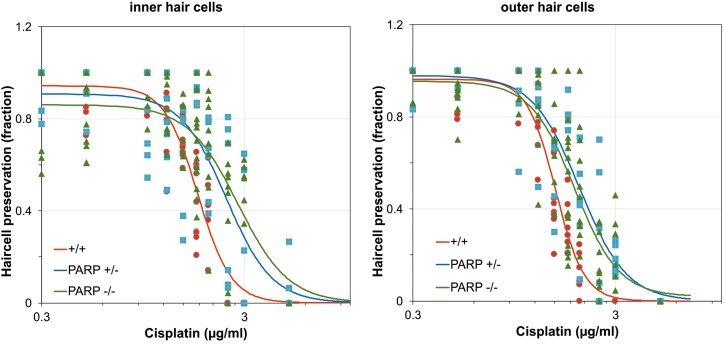
Dose-response curves after cisplatin exposure calculated for the fraction of preserved inner and outer hair cells in wildtype (red; *n* = 3–9), heterozygous (blue; *n* = 5–6), and homozygous PARP-deficient (green; *n* = 4–13) mice (11 concentrations for each genotype). Regression lines were obtained using non-linear regression analysis.

The effects of cisplatin exposure on cochleae obtained from wildtype, heterozygous, and PARP1-deficient mice in microgravity culture were quantified from whole mount preparations of the organ of Corti for control groups and cisplatin-exposure groups. Cisplatin concentrations ranged from 0.1 μg/ml to 5 μg/ml. Hair cell preservation was calculated as the fraction of cochlear length with less than 10% hair cell loss. The EC_50_ values of inner hair cell preservation were 1.77 μg/ml for wildtype, 2.45 μg/ml for heterozygous, and 2.79 μg/ml for PARP1 deficient cochleae (Figure 1). For outer hair cell preservation, the EC_50_ values for the three different genotypes were 1.52 μg/ml, 1.998 μg/ml, and 1.89 μg/ml, respectively (Figure 1). The hillslope was also affected by the genotype. The steepness of the fitted curves (hillslope) was 5.3 for the inner hair cells and 6.2 for the outer hair cells of wildtype cochleae. The hillslope values were 3.85 and 3.73 for inner and outer hair cells of heterozygous animals, respectively, and 3.48 and 3.98 for homozygous animals, respectively.

The cisplatin tolerance of cochlear hair cells was significantly higher in heterozygous and homozygous animals compared to wildtype (Table 1). When cisplatin concentration exceeded a concentration of 1.75 μg/ml, a statistically significant increase in hair cell preservation rate was observed in both heterozygous and homozygous PARP1-deficient genotypes (Table 1). For outer hair cells, nearly complete preservation (0.9) in wildtype cochleae was maintained up to a cisplatin concentration of 1 μg/ml. A complete loss of outer hair cells was observed when the concentration exceeded 2 μg/ml cisplatin. The tolerance of outer hair cells to cisplatin in heterozygous animals increased compared to wildtype cochleae (Table 1), for example using 1.75 μg/ml cisplatin, outer hair cell preservation increased from 0.33 (±0.1) to 0.81 (±0.05). Similar results were achieved for inner hair cells, here the fraction of hair cell preservation increased from 0.44 (±0.16) in wildtype animals to 0.93 (±0.06) and 0.69 (±0.19) in heterozygous and homozygous animals, respectively.

**TABLE 1:** Mean cochlear perservation (arithmetic mean, fraction) at different cisplatin concentrations in the different genotypes.

	**WiIdtype**			**PARP+/−**				**PARP−/−**			
**Cisplatin (μg/ml)**	**Mean (%)**	***SD* (%)**	***n***	**Mean (%)**	***SD* (%)**	***n***	***p* vs. wt**	**Mean (%)**	***SD* (%)**	***n***	***p* vs. wt**
**IHC**											
0.1	94.1	6.5	6	100.0	0.0	6	0.262	93.2	8.4	8	0.960
0.3	100.0	0.0	5	93.5	9.3	6	0.667	80.9	19.3	6	0.072
0.5	90.1	10.6	6	92.8	9.1	6	0.907	79.3	14.8	10	0.226
1	93.7	8.9	3	78.7	17.4	6	0.268	91.7	9.3	6	0.975
1.25	76.3	15.0	6	75.5	16.5	6	0.995	84.5	12.7	7	0.568
1.5	69.7	8.0	9	61.6	24.0	5	0.576	74.6	11.8	7	0.773
1.75	43.9	15.7	9	93.1	5.6	6	**<0.001**	69.4	18.8	13	**0.003**
2	31.1	22.1	8	62.2	15.9	6	**0.039**	69.5	24.0	10	**0.004**
2.5	0.0	0.0	7	42.4	32.9	6	**0.011**	42.2	23.1	9	**0.005**
3	0.0	0.0	7	24.3	27.5	6	**0.048**	51.7	9.4	6	**0.002**
5	0.0	0.0	3	5.5	9.7	6	0.487	0.0	0.0	4	–
Sum			69			65				86	
**OHC**											
0.1	100.0	0.0	6	100.0	0.0	6	1.000	97.1	3.0	8	0.033
0.3	100.0	0.0	5	97.2	6.2	6	0.658	95.4	6.5	6	0.347
0.5	88.4	8.7	6	98.8	2.8	6	0.070	91.1	8.7	10	0.733
1	92.3	10.8	3	88.9	15.6	6	0.896	94.8	7.6	6	0.945
1.25	75.3	13.1	6	84.9	16.5	6	0.568	81.3	19.1	7	0.781
1.5	46.9	16.3	9	55.9	15.6	5	0.604	68.3	19.1	7	**0.062**
1.75	32.8	9.8	9	80.9	5.4	6	**<0.001**	50.7	25.7	13	**0.082**
2	9.5	9.0	8	42.4	19.1	6	**0.012**	51.8	23.6	10	**<0.001**
2.5	0.0	0.0	7	27.7	27.5	6	**0.102**	17.8	11.9	9	**0.019**
3	0.0	0.0	7	18.0	5.8	6	**<0.001**	23.3	14.2	6	**<0.001**
5	0.0	0.0	3	0.0	0.0	6	–	0.0	0.0	4	–

*The standard deviation (SD), the number of observations (*n*) and *p* values of the statistical test (*p* vs. wt: Dunnett test with wildtype as control) are given. Significant values are shown in bold.*

### Inhibition of PARP Activity Through Cisplatin Treatment

**FIGURE 2:**
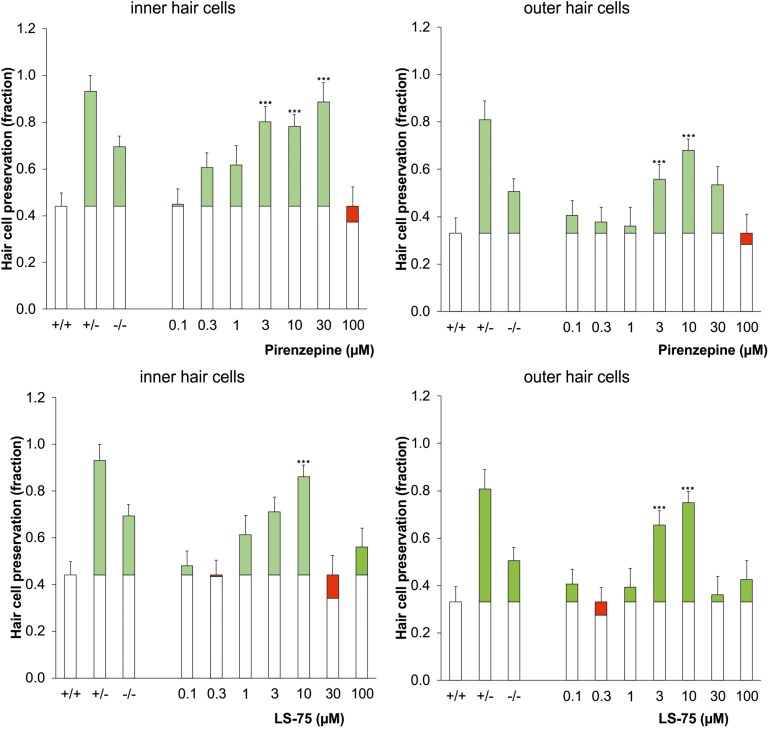
Dose-response effect of the PARP inhibitor pirenzepine (top row) or LS-75 (bottom row) on the survival of inner (left) and outer hair cells (right) in the cisplatin ototoxicity model, mimicking the effect of the genetic ablation of PARP in a dose range of 3–10 μM. As controls the genotypic results (cf. Figure 1) are plotted on the left columns. The white bars indicate the hair cell preservation rate of control cochleae, and the colored portions of the bars indicate the difference in the wildtype control (cisplatin treated) (green: preservation; red: loss). Differences to the wildtype control were calculated using Dunnetts test (^∗∗∗^*p* < 0.001 values were considered significant).

Pharmacological inhibition of PARP was tested to verify the increased cisplatin tolerance of hair cells observed in the PARP-deficient genotypes. Using 1.75 μg/ml cisplatin, wildtype cochleae were treated with the PARP inhibitors pirenzepine or LS-75 for 24 h at concentrations ranging from 0.1 to 100 μM (Figure 2 and Table 2). Cultures treated with pirenzepine or LS-75 at low concentrations of 0.1–1 μM showed poor hair cell preservation comparable to control levels (ears treated with cisplatin only). Cultures treated with Pirenzepine or LS-75 at intermediate concentrations of 3–30 μM showed (Figure 2 and Table 2 for statistics) increased hair cell preservation. Applying pirenzepine increased preservation rates for inner hair cells significantly (*p* < 0.05, Dunnett’s test) from 0.44 (±0.15) to 0.80 ± 0.11 (3 μM), 0.78 ± 0.12 (10 μM), and 0.88 ± 0.09 (30 μM). Similar observations were made for both, inner and outer hair cells. The hair cell preservation rate in cultures that were treated with 100 μM pirenzepine was unchanged compared to controls (*p* > 0.05, Dunnett’s test) compared to controls.

**TABLE 2:** Mean cochlear perservation (arithmetic mean, fraction) at 1.75 μg/ml cisplatin combined with Pirenzepin or LS-75 at various concentrations.

	**1.75 μg/ml Cisplatin**					**1.75 μg/ml Cisplatin and Pirenzepine**					**1.75 μg/ml Cisplatin and LS-75**			
	**Genotype**	**Mean**	**± *SD***	***n***	***p* vs. wt**		**Mean**	**± *SD***	***n***	***p* vs. wt**	**Mean**	**± *SD***	***n***	***p* vs. wt**
**IHC**														
	PARP −⁣/⁣−	0.69	0.19	13	**0.003**	0.1	0.45	0.11	5	1.000	0.48	0.05	3	0.999
	PARP ±	0.93	0.06	6	**<0.001**	0.3	0.61	0.13	5	0.227	0.44	0.12	4	1.000
	Wildtype	0.44	0.16	9		1	0.62	0.16	3	0.329	0.61	0.17	3	0.285
	Sum			28		3	0.80	0.11	5	**<0.001**	0.71	0.04	2	0.074
						10	0.78	0.12	8	**<0.001**	0.86	0.10	9	**<0.001**
						30	0.89	0.10	3	**<0.001**	0.34	0.14	2	0.908
						100	0.37	0.06	3	0.979	0.56	0.14	6	0.428
						Sum			32				29	
**OHC**														
	PARP −⁣/⁣−	0.51	0.26	13	0.083	0.1	0.41	0.20	3	0.889	0.40	0.09	3	0.921
	PARP ±	0.81	0.05	6	**0.000**	0.3	0.38	0.07	4	0.987	0.28	0.08	4	0.979
	Wildtype	0.33	0.10	9		1	0.36	0.03	3	1.000	0.39	0.07	3	0.965
						3	0.56	0.16	2	**0.031**	0.66	0.12	2	**0.010**
						10	0.68	0.14	9	**<0.001**	0.75	0.13	9	**<0.0.001**
						30	0.54	0.06	2	0.156	0.36	0.06	2	1.000
						100	0.28	0.05	6	0.998	0.43	0.17	6	0.561

*The standard deviation (SD), the number of observations (*n*) and *p* values of the statistical test (*p* vs wt: Dunnett test with wildtype as control) are given. Significant values are shown in bold.*

Using LS-75, preservations of inner and outer hair cells rates were significant (*p* < 0.05, Dunnett’s test) different from those in wild type mice at 3 and 10 μM, reaching maxima at 0.75 ± 0.04 for the outer hair cells and 0.86 ± 0.06 for the inner hair cells at 10 μM respectively. The hair cell preservation rate in cultures that were treated with 30 or 100 μM LS-75 was unchanged (*p* < 0.05, Dunnett’s test) compared to controls.

### Cytostatic Potency of Cisplatin Is Not Reduced by PARP Inhibitors

**FIGURE 3:**
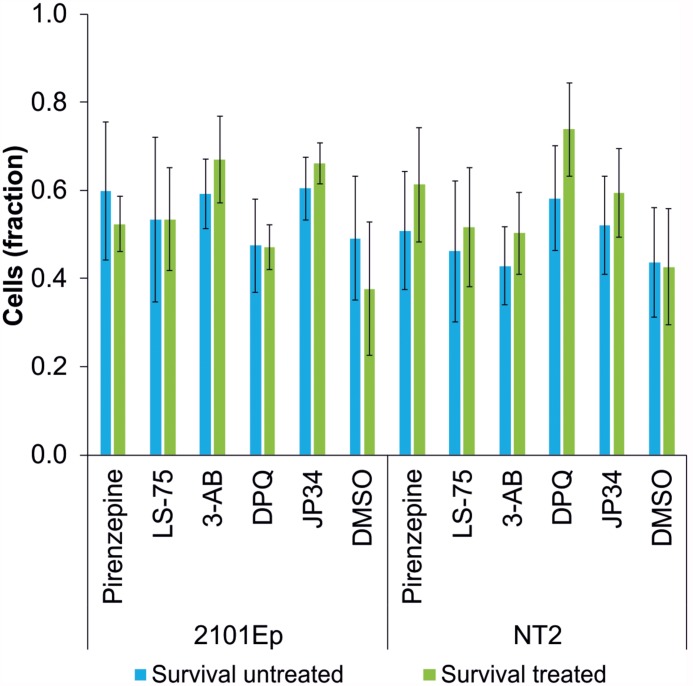
Survival rate of cisplatin-treated tumor cell lines (2102Ep and NT2 germ cell tumor lines) without (blue bars) and with treatment (green bars) of PARP inhibitors (Pirenzipine, LS-75: 10 μM; 3-AB: 100 μM; DPQ, PJ34: 1 μM; DMSO: 0.1%). No statistically significant difference in survival rate was observed. Data are expressed as the mean (*n* = 4).

To study the influence of PARP inhibitors on the cytostatic potency of cisplatin, the effects of different PARP inhibitors on the survival of cisplatin-treated tumor cells was tested. The germ cell tumor lines 2102Ep and NT2 (Podrygajlo et al., 2009) were treated with 1.4 μM cisplatin and the PARP inhibitors LS-75 (10 μM), pirenzepine (10 μM), 3-AB (100 μM), DPQ (1 μM), and PJ34 (1 μM). No effect on the survival of cells was observed for any of the PARP inhibitors investigated (Figure 3).

## Discussion

In this study, we used a genetic mouse model and demonstrated that PARP deficiency exhibits an otoprotective effect by increasing the tolerance to cisplatin exposure. We also showed that the effect observed in the genetic model was reproduced pharmacologically in applying the PARP-inhibitor pirenzepine or its metabolite LS-75. We furthermore extend upon our previous studies (Hahn et al., 2008; Arnold et al., 2010; Tropitzsch et al., 2014) regarding the use of a rotating bioreactor system that allows for successful culture of the whole inner ear organ.

Several models of cisplatin ototoxicity in mammals have been introduced. Previati et al. (2007) used an immortalized cell line (OC-k3 cells) derived from the organ of Corti of transgenic mice (Immortomouse^TM^). Other experiments of cochlear and utricular organotypic cultures were performed on preparations obtained from postnatal day 3–4 rats (Zhang et al., 2003). Similarly, Yarin et al. (2005) used organotypic cultures of the organ of Corti from 3- to 5-day-old rats, while earlier studies used organ of Corti explants from 3-day-old rats (Liu et al., 1998). However, the otoprotective effect of PARP1 inhibition has thus far only been described in models of ischemia-reperfusion (Tabuchi et al., 2001) and acoustic trauma (Tabuchi et al., 2003) *in vivo*, but not in the context of cisplatin ototoxicity.

The primary biological target of cisplatin is DNA (Reviews: (Jamieson and Lippard, 1999; Wang et al., 2004), distorting the DNA duplex. Subsequently, cell death occurs through apoptosis and necrosis via several complex signal pathways. In primary cultures of mouse proximal tubular cells, apoptotic and necrotic cell death is dependent on cisplatin concentration. High concentrations of cisplatin (800 μM) led to necrotic cell death over a few hours, whereas much lower concentrations of cisplatin (8 μM) led to apoptosis (Lieberthal et al., 1996). The study furthermore concluded that reactive oxygen species played a role in mediating apoptosis, but not necrosis, induced by cisplatin.

DNA damage repair is mediated to a large extent by the PARP1 enzyme (De Vos et al., 2012), which is generally considered as a factor beneficial for cell physiology. However, in cancer, PARP-mediated repair of DNA damage allows cells to survive and can potentially contribute to cancerogenesis. Therefore, numerous PARP inhibitors have been developed clinically to be used as adjuvant and maintenance therapies, together with DNA damaging agents, including cisplatin (Ledermann, 2016). Consequently, extensive human tolerability and efficacy data are already available (Sonnenblick and Piccart, 2015). Paradoxically, overactivation of PARP is also associated with increased cell death, primarily in postmitotic, non-dividing cells and tissues (Paquet-Durand et al., 2007; Wang et al., 2011). This may explain why, in the post-mitotic cells of the inner ear and also in the retina (Sahaboglu et al., 2016), PARP inhibitors can have beneficial, pro-survival effects, while in dividing cancerous cells, loss of PARP activity increases cell death. In this context, it is interesting to note that the heterozygous organ cultures (i.e., PARP1^+/–^) showed a better hair cell preservation than the full knockout cultures, indicating that some level of PARP activity is still required for their survival. This would then correspond to the effects of pharmacological PARP inhibition where it is likely that PARP was only partially inhibited, and high inhibitor concentrations eventually showed a detrimental effect. Interestingly, overactivation of PARP may also be involved in the pathology of kidney damage where the PARP inhibitor 3-AB protected from acute kidney injury after septic shock (Wang et al., 2018).

Pirenzepine is a topical antiulcerative M1 muscarinic antagonist that inhibits gastric secretion (Carmine and Brogden, 1985). It promotes the healing of duodenal ulcers and due to its cytoprotective action is beneficial in the prevention of duodenal ulcer recurrence. It is generally well tolerated by patients. To date, pirenzepine does not appear to have been tested for possible beneficial effects on hearing and to the best of our knowledge this is the first study to show that pirenzepine can offset the ototoxic effects of cisplatin chemotherapy. Remarkably, pirenzepine is known to have a marked effect on the retina, leading to a decrease in eye length growth and preventing the progression of myopia in children and adolescents (Smith and Walline, 2015). In the retina other selective PARP inhibitors, such as PJ34 and olaparib, were found to have protective effects on photoreceptor degeneration (Paquet-Durand et al., 2007; Sahaboglu et al., 2010, 2016). On a similar level of evidence, in the inner ear, PARP activation was associated with the degeneration and loss of hair cells (Jaumann et al., 2012). Together, these studies support the concept of the use of PARP inhibitors to protect postmitotic sensory cells.

In conclusion, the PARP1-deficient model revealed a protective effect against cisplatin treatment on hair cell survival. This effect was confirmed using the PARP inhibitor pirenzepine and its metabolite LS-75, which protected against cisplatin ototoxicity in a dose-dependent manner. Additionally, the cytostatic potential of cisplatin was not hampered in two germ cell tumor lines, making PARP an attractive target for an adjuvant therapy aimed at limiting the negative ototoxic side-effects of cisplatin anti-cancer treatments.

## Data Availability

All datasets generated for this study are included in the manuscript and/or the supplementary files.

## Ethics Statement

Animal use for organ explants was approved by the Committee for Animal Experiments of the Regional Council (Regierungspräsidium) of Tübingen.

## Author Contributions

AT, MM, FM, and AM performed the experiments. AT, MM, H-GK, and HL analyzed the data. MM, H-GK, AS, and HL conceived the study. AT, MM, FP-D, AM, and HL wrote and revised the manuscript.

## Conflict of Interest Statement

ProteoSys AG provided the compounds pirenzepine and LS-75 for the study. Based on the results attained by the research funded by ProteoSys AG and the compounds provided by ProteoSys AG, a patent (Schrattenholz et al., 2010) was filed by and granted to ProteoSys AG (Inventor Andre Schrattenholz; Title: Pirenzepine as otoprotective agent. Publication number: 20110263574, October 27, 2011). AS was CSO and shareholder of ProteoSys AG. As part of the contract research agreement, ProteoSys AG has granted potential milestone payments and royalties to the University of Tübingen Medical Center in the event of commercialization of ProteoSys AG. AS deceased in 2014. The funder had no role in the study design, data collection or publication of the manuscript. HL is shareholder of Acousia Therapeutics GmbH, Tübingen, Germany.

The remaining authors declare that the research was conducted in the absence of any commercial or financial relationships that could be construed as a potential conflict of interest.
